# Curcumin-Loaded mPEG-PLGA Nanoparticles Attenuates the Apoptosis and Corticosteroid Resistance Induced by Cigarette Smoke Extract

**DOI:** 10.3389/fphar.2022.824652

**Published:** 2022-02-25

**Authors:** Xi Chen, Di Wang, Xuejun Guo, Xiaoming Li, Wenjing Ye, Yang Qi, Wen Gu

**Affiliations:** ^1^ Department of Respirology Medicine, Xinhua Hospital, School of Medicine, Shanghai Jiaotong University, Shanghai, China; ^2^ Department of Intensive Care Medicine, The First Affiliated Hospital of USTC, Division of Life Sciences and Medicine, University of Science and Technology of China, Hefei, China; ^3^ Department of Pharmacy, No. 900 Hospital of the Chinese PLA Joint Support Forces, Fuzhou, China

**Keywords:** curcumin, chronic obstructive pulmonary disease, apotosis, IL-8, HDAC2

## Abstract

The present study was aim to prepare curcumin-loaded methoxypolyethylene-glycols-poly (lactic-co-glycolic acid) nanoparticles (Cur-mPEG-PLGA-NPs) and investigate curcumin’s effect on reversing corticosteroid resistance induced by cigarette smoke extract (CSE) in rat tracheal epithelial (RTE) cells. The Cur-mPEG-PLGA-NPs were spherical, regular in shape with smooth surfaces, and well distributed and Cur-mPEG-PLGA-NP suspensions had good water solubility and presented prolonged release. Furthermore, we found that Cur-mPEG-PLGA-NPs were internalized more than curcumin into the cells and significantly alleviated apoptosis in RTE cells. In addition, 10% CSE reduced the maximal inhibition percentage and increased the half-inhibitory concentration of budesonide (BUD) on IL-8 secretion, and curcumin restored the efficacy of BUD inhibition. BUD in combination with Cur-mPEG-PLGA-NPs showed higher inhibitory rates for LPS- and CSE-induced IL-8 secretion than that in combination with curcumin. Moverover, the relative expression levels of HDAC2 was reduced after CSE exposure and curcumin could improve HDAC2 expression and reverse CSE-induced corticosteroid resistance. Curcumin in high concentration and Cur-mPEG-PLGA-NPs restored HDAC2 levels in RTE cells and thus Cur-mPEG-PCL-NPs have higher biological activity than curcumin.

## Introduction

Chronic obstructive pulmonary disease (COPD) is characterized by lung inflammation, airflow obstruction, and tissue destruction (Ito et al., 2005; Zhang et al., 2020). COPD is an aging disease with high prevalence and mortality. The aging of an ever-expanding world population, air pollution in the developing world, and high rates of smoking ensure that COPD will remain a global challenge in the 21st century (Lopez-Campos et al., 2016).The current drug management of COPD includes bronchodilators (muscarinic receptor antagonists and β2-agonists) associated with inhaled corticosteroids (ICSs), especially in patients with frequent exacerbations or severe COPD. However, in contrast to asthmatic patients, COPD patients are low and unresponsive to even high doses of ICSs. ICSs have less effect on controlling the decline in lung function and suppressing chronic inflammation, leading to the development of corticosteroid resistance (Jiang and Zhu, 2016).

The molecular mechanisms of corticosteroid resistance in COPD are better understood now. A balance between histone acetyltransferase (HAT) and histone deacetylase (HDAC) is crucial for a normal inflammation reaction and effective inflammation suppression, and HDAC2-activity decrease is a contributing factor in corticosteroid resistance (Lai et al., 2018). In pro-inflammatory responses, activated NF-kappaB forms a protein complex with HAT, leading to histone acetylation and pro-inflammatory gene transcription. GR-alpha, one of 2 GR isoforms, can increase HDAC2 recruitment, resulting in deacetylation of histones in the promoter region of key pro-inflammatory cytokines, leading to inhibition of inflammation (Barnes, 2013b). Hence, modulating the activity of HDAC2 is a potential therapeutic approach to overcome corticosteroid resistance.

A variety of reagents such as phosphoinositide-3-kinase (PI3K) inhibitors and sulforaphane have been demonstrated to improve corticosteroid sensitivity by restoring the activity and expression of HDAC2 (Chen et al., 2012; Barnes, 2013a) . However, these agents are liable to cause systemic side effects and have a narrow therapeutic index. Therefore, developing drugs with only minor side effects and an ability to regulate HDAC2 activity has recently become the research focus for the reversal of corticosteroid resistance.

Curcumin (Cur), derived from the plant *Curcuma longa*, is used as a medicinal agent in many parts of the world, especially the Indian subcontinent. It has been reported that curcumin can restore HDAC2 protein levels by scavenging oxygen free radicals induced by cigarette smoke extract (CSE) and, hence, reverse corticosteroid resistance (Soflaei et al., 2018). Curcumin modulates the histone acetylation and deacetylation process in the promoter region of the pro-inflammatory cytokine gene, leading to reversal of corticosteroid resistance in type II alveolar epithelial cells (Ito et al., 2006). In addition, curcumin has the therapeutic potential to reverse corticosteroid resistance in COPD patients (Osoata et al., 2009). Curcumin has been extensively studied owing to its wide range of biological activities, which include anti-inflammatory, anti-infective, anti-arthritic, anti-cancer, and wound-healing potentials. Nevertheless, the clinical application of curcumin is limited, owing to its poor aqueous solubility, chemical instability, low oral bioavailability, inadequate absorption, rapid metabolism and elimination, and transmembrane permeation (To et al., 2010).

In recent years, nanotechnology-based delivery systems have been developed to overcome the pharmaceutical issues related to the delivery of therapeutic agents. Advantages such as avoiding enzymatic degradation of drugs, providing controlled release of therapeutic payloads, enhancing water solubility, improving cellular uptake, and optimizing target-specific delivery have made nanoparticles widely recognized (Umerska et al., 2018). At present, nanotechnology-based delivery carriers include liposomes, polymeric NPs, nanocomposite hydrogels, nanohybrid scaffolds, solid lipid nanoparticles, nanostructured lipid carriers, and nanofibers (Mohanty and Sahoo, 2010; Ungaro et al., 2012). Polymeric NPs have received extensive attention owing to some distinct properties, such as their ultra-small size, high encapsulation efficiency, adequate surface potential, bio-degradability, and bio-compatibility (Zhang et al., 2014). Many studies have demonstrated that polymeric NPs can overcome the physical and chemical shortcomings of curcumin and improve its bioavailability. A polymeric NP delivery system improves the anti-oxidant, anti-inflammatory, and anti-tumor performance of curcumin (Yang et al., 2007). In addition, application of curcumin polymeric NPs as complementary agents is perfect for reducing the dose of the main therapeutic agent, resulting in enhanced therapeutic efficacy while reducing systemic toxicity (Umerska et al., 2018).

Therefore, we prepared Cur-mPEG-PLGA-NPs via emulsion solvent evaporation, to overcome the physical and chemical disadvantages of curcumin and improve its therapeutic activity. In addition, the effect of curcumin on reversing corticosteroid resistance induced by CSE was also investigated and biological activities between curcumin and Cur-mPEG-PLGA-NPs were investigated.

## Materials and Methods

### Reagents and Materials

mPEG-PLGA (PLGA, DL-lactide-co-glycolide, 50/50 Poly, MW 20 kDa; mPEG, MW 2 kDa) was purchased from Daigang Biological Science and Technology Co.,, Ltd., (Shandong, China). Curcumin (purity ≥98%) and vitamin E-TPGS were purchased from Aladdin Industrial Corporation (Shanghai, China). Acetone and tetrahydrofuran (THF) were purchased from Sinopharm Chemical Reagent Co., Ltd., (Shanghai, China). Rat tracheal epithelial (RTE) cells were purchased from the Cell Culture Center of the Chinese Academy of Medical Sciences (Beijing, China). Budesonide (BUD) was purchased from AstraZeneca PLC (London, United Kingdom). β-actin (catalog #4970) antibody and HDAC Antibody Sampler Kit (catalog#9928) were bought from Cell Signaling Technology (Beverly, MA, United States).

### Preparation of Nanoparticles

mPEG-PLGA nanoparticles containing curcumin (Cur-mPEG-PLGA-NPs) were synthesized via the emulsion solvent evaporation method. At room temperature, 30 mg mPEG-PLGA and 1 mg curcumin were dissolved in 3 ml acetone-ethanol solvent (volume ratio 1:1) to form an organic phase. The organic phase was slowly dropped into the PVA solution under magnetic stirring (300 rpm), which was continued for 5 min, to form a nanoparticle suspension. The acetone-ethanol solvent was removed via vacuum rotary evaporation for 40 min, and large particles were removed via centrifugation at 2000 rpm for 20 min. The nanoparticles were precipitated via centrifugation at 12,000 rpm for 30 min, and the supernatant was discarded to remove residual PVA and mPEG-PLGA. The residue was the colloidal suspension of Cur-mPEG-PLGA-NPs.

### Characterization of Nanoparticles

The absorption wavelength of curcumin and standard curve of curcumin concentration were defined. mPEG-PLGA and curcumin were dissolved in THF, and then scanned using a Dinuolitai UV-Vis3000 spectrophotometer (Shanghai, China) in the wavelength of 200–600 nm with blank solvent as the reference. A standard curve correlating absorbance at 423 nm with the concentration of curcumin was created. The standard curve equation was used to calculate the concentration of curcumin or curcumin loaded into mPEG-PLGA nanoparticles in following studies.

Weight of drug feeded (Wt) was obtained by measuring total curcumin in a nanoparticle suspension. Another nanoparticle suspension was accelerated freezing centrifuged for 40 min at 20,000 rpm and weight of drug free (Wf) was obtained by measuring curcumin in the supernatant. Weight of feeding polymer and drug (Ws) was the total amount of drug and carrier added to the preparation. Encapsulation efficiency (EE) = (Wt − Wf)/Wt; drug loading (DL) = (Wt − Wf)/Ws.

The size distribution spectra, zeta potential, and morphology were determined using a HORIBA LB550 laser scattering particle size analyzer (Japan), Malvin zeta potentiometer (Malvern, United Kingdom), and transmission electron microscope (TEM), respectively.

### *In vitro* Release of Curcumin From Nanoparticles

The *in vitro* drug release behavior of Cur-mPEG-PLGA-NPs was determined using the dynamic dialysis method. A nanoparticle suspension was loaded into a pre dialysis bag, both ends of which were clamped. Then, the bag was hung in a brown bottle containing 200 ml 0.5% SDS in water solution at (37.0 ± 0.5)°C with gentle shaking. At specific time points, 4 ml samples were collected, and 4 ml release medium was added. SDS in water solution (0.5%) was used as the blank control, and curcumin absorbance was measured at 423 nm. The concentration of curcumin was calculated according to the standard curve, and the cumulative drug release rate was determined.

### Analysis of Cytotoxicity, Apoptosis and LDH Release Assay

The RTE cell line, obtained from GuangZhou Jennio Biotech Co., Ltd., was cultured in minimal essential medium with Earle’s salts (MEM-EBSS, Life Technologies) supplemented with 20% fetal calf serum (HyClone; Thermo Scientific), 100 U/mL penicillin, and 100 mg/ml streptomycin in a 5% CO_2_ humidified atmosphere at 37°C. RTE cells in the logarithmic growth phase were inoculated into a 96-well plate at a density of 5 × 10^3^ cells/well and cultured for 24 h. Cells were then treated with curcumin of different concentrations (5 μM, 10 μM, 15 μM, 20 μM, 25 μM) or Cur-mPEG-PLGA-NPs (at doses equivalent to the concentration gradients of curcumin) and sequentially cultured for 48 h. The cell viability rate was determined by CCK8 assay according to the recommended protocol (Promega). After digestion with trypsin, the cells were collected, washed with PBS and resuspended by 1× Binding buffer. Annexin V-FITC and solution were added and react at room temperature for 15 min, then detected by flow cytometry (FACSCalibur, BD) within 1 h. LDH assay was performed by a commercial kit (Promega, Madison, WI, United States) according to the manufacturer’s protocol.

### Quantitative Cellular Uptake Study

The cellular uptake of curcumin and Cur-mPEG-PLGA-NPs was determined by following the method of Chandana et al. (Prasad et al., 2014). RTE cells were inoculated into 24-well plates at densities of 5×10^4^ cells/well in 1 ml growth medium. The cells were incubated for 24 h at 37°C for adhesion. Then, the cells were treated with curcumin (100 nM) and Cur-mPEG-PLGA-NPs at a dose equivalent. After being incubated for different periods, the cells were washed twice with PBS to remove free drug molecules. Subsequently, the cells were lysed with methanol. The cell lysates were centrifuged at 10,000 rpm for 20 min at 4°C. Absorbance of curcumin in the supernatant of the two groups was measured and curcumin content was calculated.

### Qualitative Cellular Uptake Study

The qualitative uptake of curcumin and Cur-mPEG-PLGA-NPs in RTE cells was detected by confocal microscopy. Cells were inoculated in a petri dish at a density of 1 × 10^4^ cells/well and cultured for 24 h. Then, the cells were treated with curcumin (100 nM) and Cur-mPEG-PLGA-NPs at a dose equivalent and incubated at 37°C for different periods (2, 6, and 12 h). After incubation, the cells were fixed with 2% paraformaldehyde PBS solution for 10 min. Then, the cells were washed three times by physiological saline. Fluorescence from curcumin and curcumin loaded into mPEG-PLGA nanoparticles that entered into cells was observed using a fluorescence and light microscope with an argon laser (Olympus IX71; Olympus Corporation, Tokyo, Japan).

### Preparation of Cigarette Smoke Extract Solutions

Smoke from 1 cigarette was dissolved in 10 ml medium under negative pressure in 2 min. Subsequently, the medium was filtered through a 0.22 μm membrane, and then used as a 100% smoke extract solution (100% CSE). The CSE preparation was standardized by measuring its absorbance at 320 nm, which showed very little variation between different preparations of CSE. CSE was freshly prepared for each experiment and diluted with culture medium.

### Cell Stimulation, Intervention, and Inflammatory Mediators Assay

RTE cells in the logarithmic phase were inoculated into a 6-well plate at 2 × 10^4^ cells/well. After cell adhesion was completed, medium containing 1% FBS was used for 12 h to starve cells. Then, the cells were treated with different concentration BUD, curcumin, or curcumin + BUD for 15 min and subsequently treated with 1 μg/ml LPS and stimulated by 10% CSE for 4 h before being treated by LPS. After the cells were incubated for 24 h, the supernatant was collected.

Another set of RTE cells was cultured by the above methods. The cells were treated by 100 nM curcumin + BUD or Cur-mPEG-PLGA-NPs (100 nM)+BUD for 15 min. Then, the cells were stimulated by 10% CES for 4 h, followed by stimulation by LPS. After the cells were incubated for 24 h, the supernatant was collected.

The cell supernatant was detected according to the operational guidelines provided by ELISA kit (eBioscience) according to the manufacturer’s protocol. The inhibition rate and the half-inhibitory concentration (IC_50_) of BUD on inhibiting IL-8 secretion were calculated.

### Western Blot Analysis of HDACs

RTE cells were cultured according to the above methods. The cells were treated with BUD (100 nM), curcumin (100 nM), and Cur-mPEG-PLGA-NPs (100 nM) for 15 min and subsequently stimulated by 10% CSE. After incubation for 24 h, cells were collected and lysed by RIPA buffer. The total protein concentration of cell samples was measured by the Pierce BCA protein assay. The proteins were separated by electrophoresis on polyacrylamide gel, and then transferred to PVDF membranes. The PVDF membrane was incubated with primary antibody overnight at 4°C and secondary antibody for 120 min at room temperature. Semi-quantitative analysis was performed using ChemiDoc XRS gel imaging system (Bio-Rad, United States).

### Statistical Analysis

Results were expressed as mean ± SEM from three independent experiments. The results were statistically evaluated using by one-way analysis of variance (ANOVA) followed by the Dunnett’s test. A value of *p* < 0.05 indicates a significant difference.

## Results

### Characteristics of Cur-NPs

**FIGURE 1:**
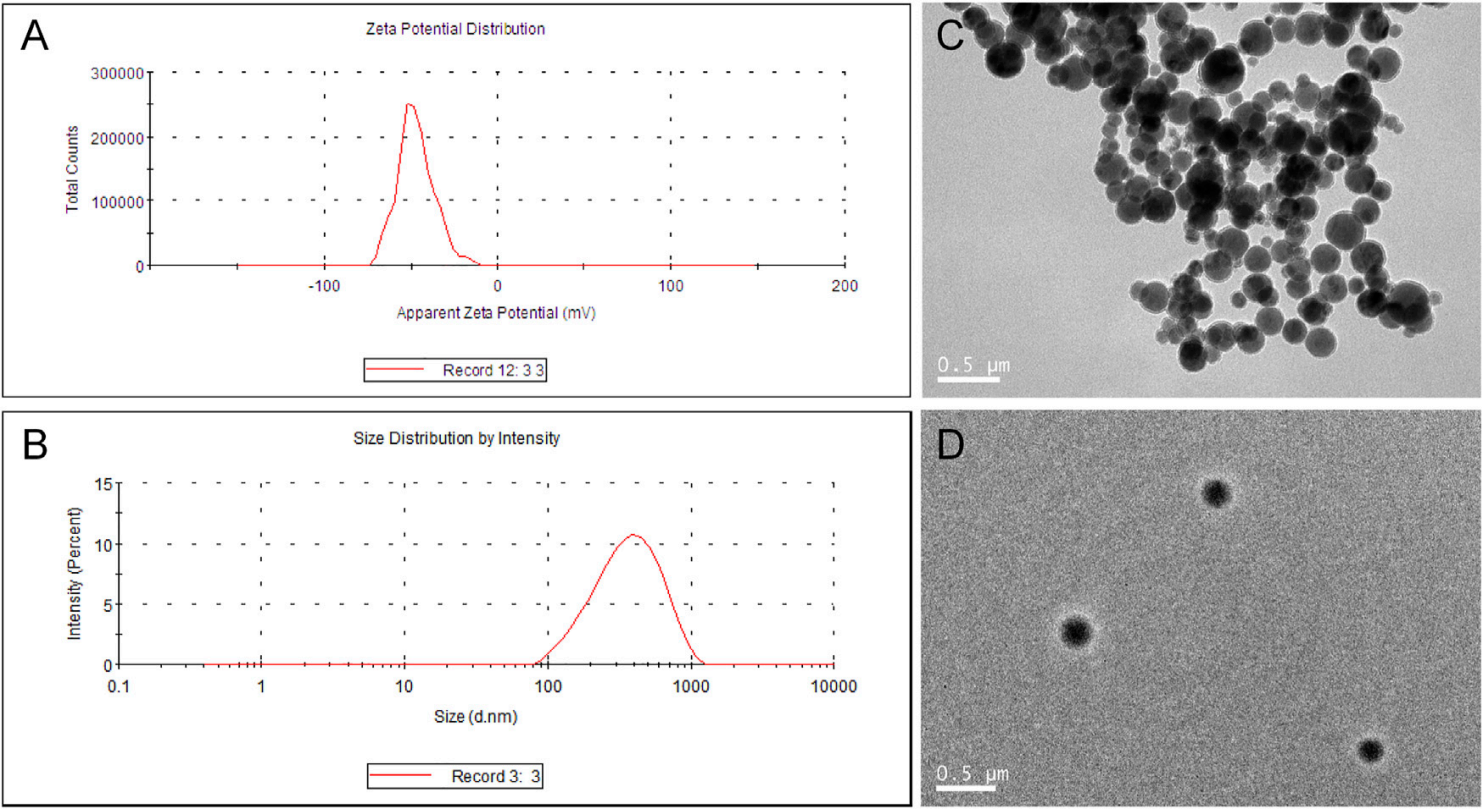
**(A)** Mean particle size of Cur-mPEG-PLGA-NPs measured by a particle size analyzer. **(B)** Zeta potential of Cur-mPEG-PLGA-NPs measured by a Malvin zeta potentiometer. **(C,D)** Morphology of Cur-mPEG-PLGA-NPs measured by a transmission electron microscope.

**FIGURE 2:**
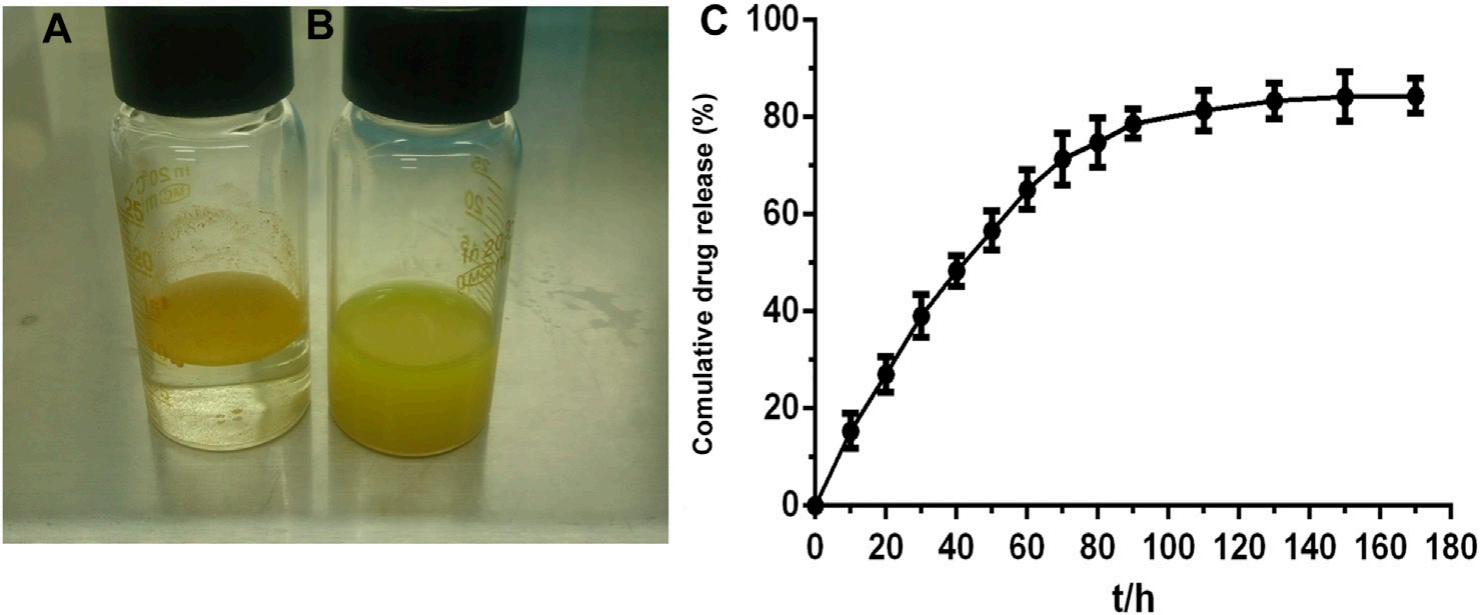
**(A)** Cur (10 mg) dissolved in PBS (0.01 M, pH 7.4) was insoluble in aqueous solution. **(B)** Cur-mPEG-PLGA-NPs at a dose equivalent to 10 mg curcumin were dissolved in aqueous solution. **(C)** *In vitro* release profile of Cur in 0.5% SDS in water solution at (37.0 ± 0.5)°C from Cur- mPEG-PLGA-NPs.

Cur-mPEG-PLGA-NPs were synthesized with encapsulation efficiency of (83.05 + 1.07)% and drug loading rate of (10.87 + 0.58)%. The negative zeta potential and the average diameter of nanoparticles were −29.4 ± 5.47 mV (Figure 1A) and 376.3 ± 2.69 nm (Figure 1B), respectively. Moreover, the Cur-mPEG-PLGA-NPs were spherical, regular in shape with smooth surfaces, and well distributed (Figures 1C,D). Curcumin had maximum absorption at 400 nm, while mPEG-PLGA had little interference with the UV absorption of curcumin at this wavelength; therefore, the detection wavelength of curcumin was approximately 423 nm. Cur-mPEG-PLGA-NPs in distilled water exhibited a homogeneous suspension (Figure 2B); in contrast, curcumin was poorly soluble in distilled water (Figure 2A). As shown in Figure 2C, approximately (84.4 ± 3.58)% of the total curcumin was released in 7 days, showing a prolonged release behavior.

### Cur and Cur-mPEG-PLGA-NPs Protected Against CSE-Induced Apoptosis

**FIGURE 3:**
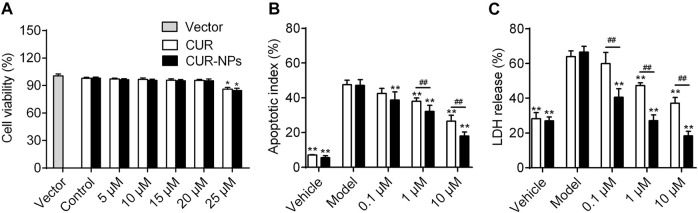
Cur and Cur-mPEG-PLGA-NPs protected against CSE-induced cytotoxicity in RTE cells. **(A)** Viability of RTE cells with various concentrations of Cur and Cur-mPEG-PLGA-NPs for 48 h using a CCK8 bioassay. **(B)** Apoptosis assay of RTE cells exposed to CSE and different concentration administration of Cur or Cur-mPEG-PLGA-NPs were examined by flow cytometry. Cells were gated for an annexin V^+^ (*x*-axis) versus PI^+^ (*y*-axis) contour plot. **(C)** Cell cytotoxicity was determined by LDH activity. ^*^ *p* < 0.01 and ^**^ *p* < 0.05 related to the control group; ^##^ *p* < 0.01 compared to CUR group.

Accordingly, 20 μM and the concentration equivalent to 20 μM curcumin were chosen as the upper limit of the intervention concentrations of curcumin and Cur-mPEG-PLGA-NPs (Figure 3A). We next determined whether Cur and Cur-mPEG-PLGA-NPs could protect against CSE-induced apoptosis (Figure 3B). After the 10% CSE injure, RTE cells were suffered significantly apoptosis. Next, we found that Cur and Cur-mPEG-PLGA-NPs attenuated these apoptotic changes (Figure 2B).

Similar findings were also obtained with LDH release assays in RTE cells exposed to 10% CSE (Figure 2C). The data indicated that Cur and Cur-mPEG-PLGA-NPs protected against CSE-induced apoptosis.

### Cellular Uptake of Curcumin Loaded in Nanoparticles

**FIGURE 4:**
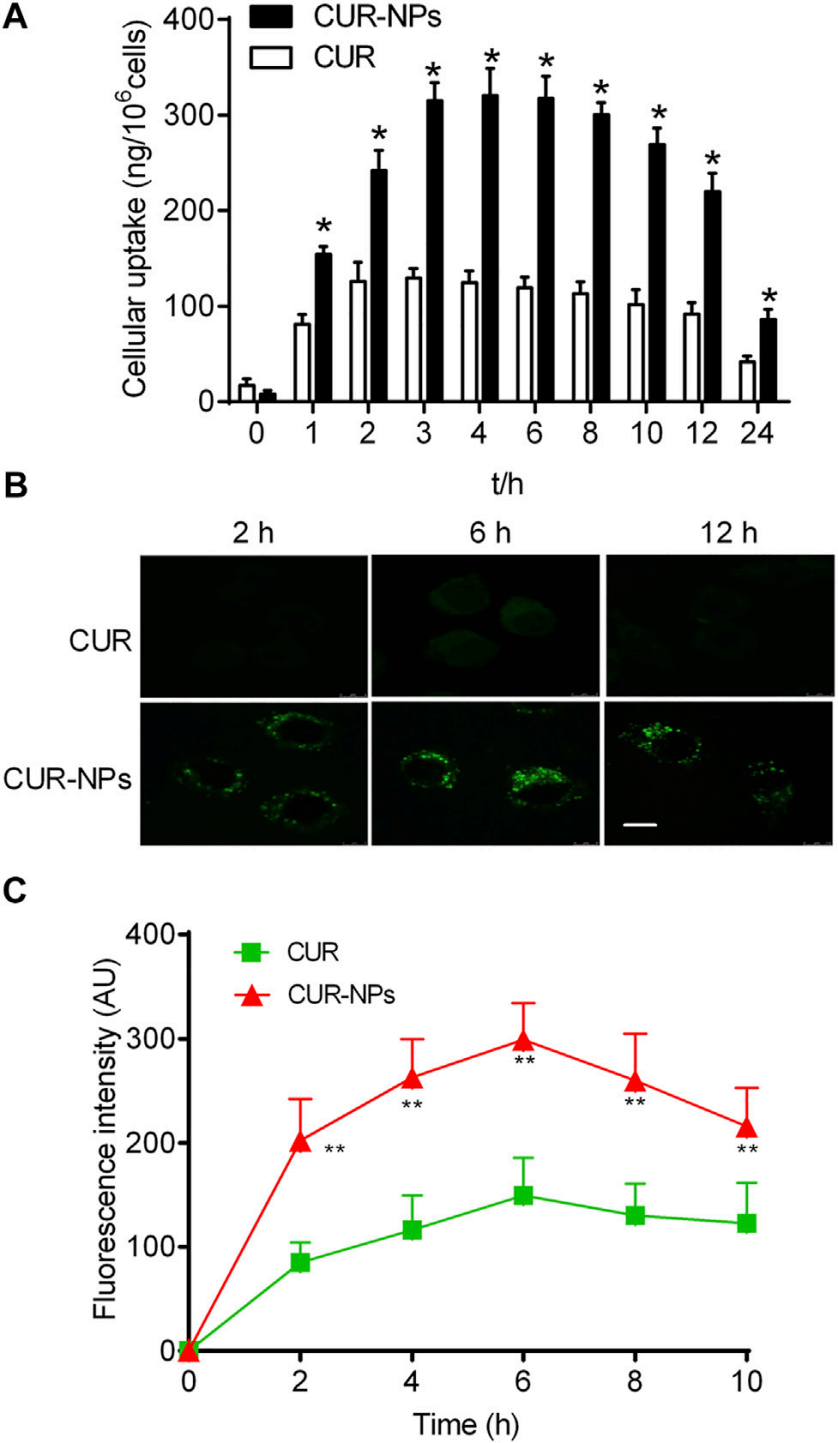
Quantitative cellular uptake study of Cur and Cur-mPEG-PLGA-NPs for different periods on RTE cells **(A)**. **(B,C)** Confocal image showing time-dependent change in intracellular fluorescence intensity of Cur-mPEG-PLGA-NPs and Cur in RTE cells. Scale bar = 5 μm ^*^ *p* < 0.05 related to cells incubated with curcumin.

Our studies showed that curcumin loaded in mPEG-PLGA-NPs was internalized more than curcumin was in RTE cells (Figure 4A). The intracellular uptake of curcumin loaded in nanoparticles reached maximum at 4 h. In contrast, the intracellular uptake of curcumin reached maximum at 3 h. The highest cellular uptake of curcumin loaded in nanoparticles was nearly 3 times that of curcumin. Moreover, our results revealed an incubation time-dependent increase of intracellular fluorescence intensity of curcumin loaded in nanoparticles in RTE cells due to sustained release of encapsulated curcumin. In contrast, a decrease in fluorescence intensity was observed for curcumin, perhaps due to a loss of stability over time (Figure 4B).

### Cur-mPEG-PLGA-NPs Inhibits LPS and CSE-Induced Inflammatory Mediators in RTE Cells

**FIGURE 5:**
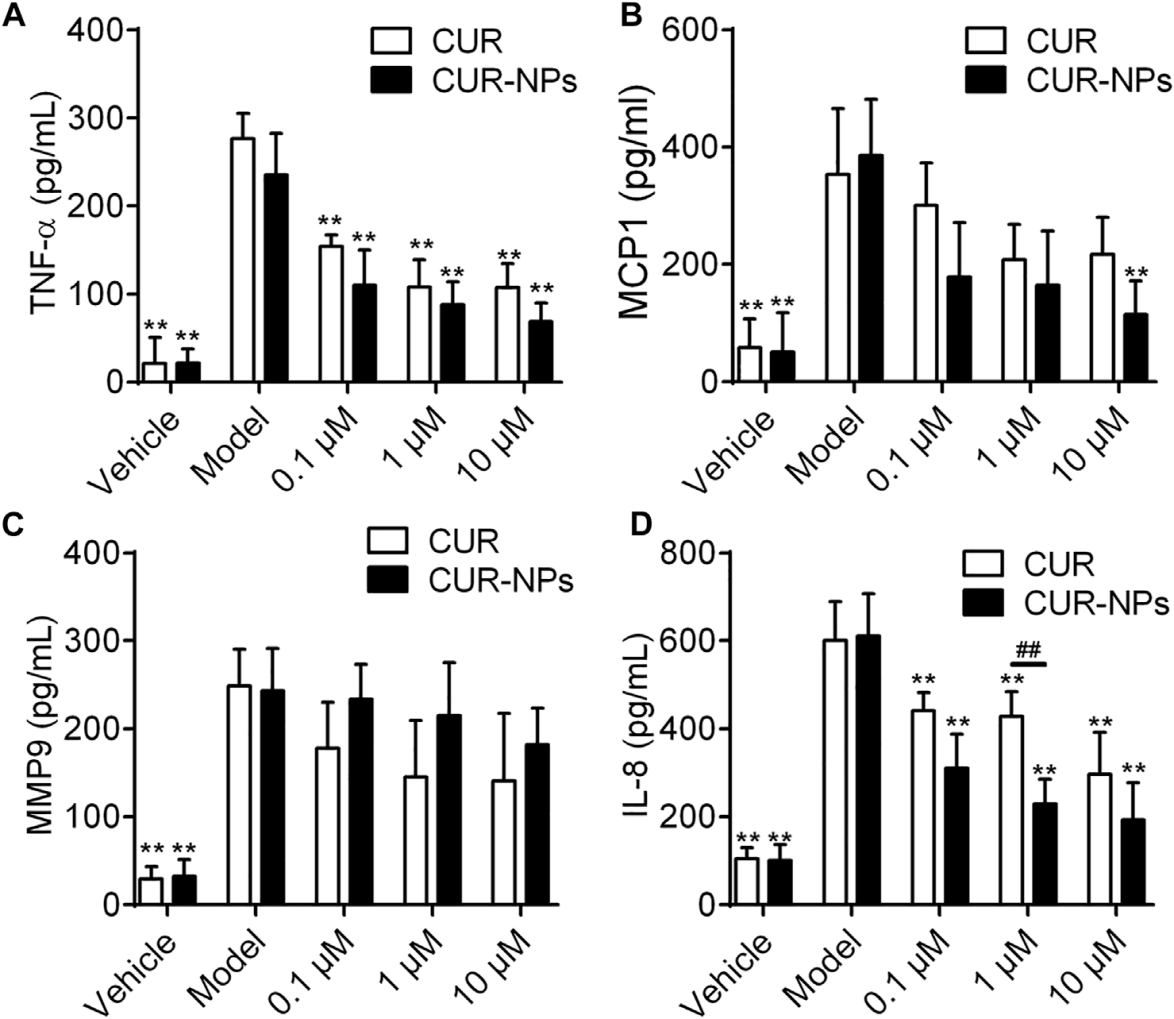
Effect of Cur and Cur-mPEG-PLGA-NPs on the production of TNF-α **(A)**, MCP-1 **(B)**, MMP-9 **(C)** and IL-8 **(D)** in CSE-injured RTE cells. RTE cells were treated with 0.1, 1, 10 μM Cur or Cur-mPEG-PLGA-NPs for 2 h, then stimulated with 10% CSE, the supernatants were collected for the assay. ^##^ *p* < 0.01 versus curcumin group, ^**^ *p* < 0.01 versus model group.

The levels of LPS and CSE-induced inflammatory mediators in RTE cells were detected by ELISA (Figure 5). We found that compared with the control group, the levels of TNF-α, MCP-1, MMP-9 and IL-8 in LPS + CSE group were significantly increased and Cur-mPEG-PLGA-NPs inhibits LPS and CSE-induced TNF-α, MCP-1 and IL-8 production in RTE Cells.

### Effect of Curcumin on BUD Resistance Induced by CSE

**FIGURE 6:**
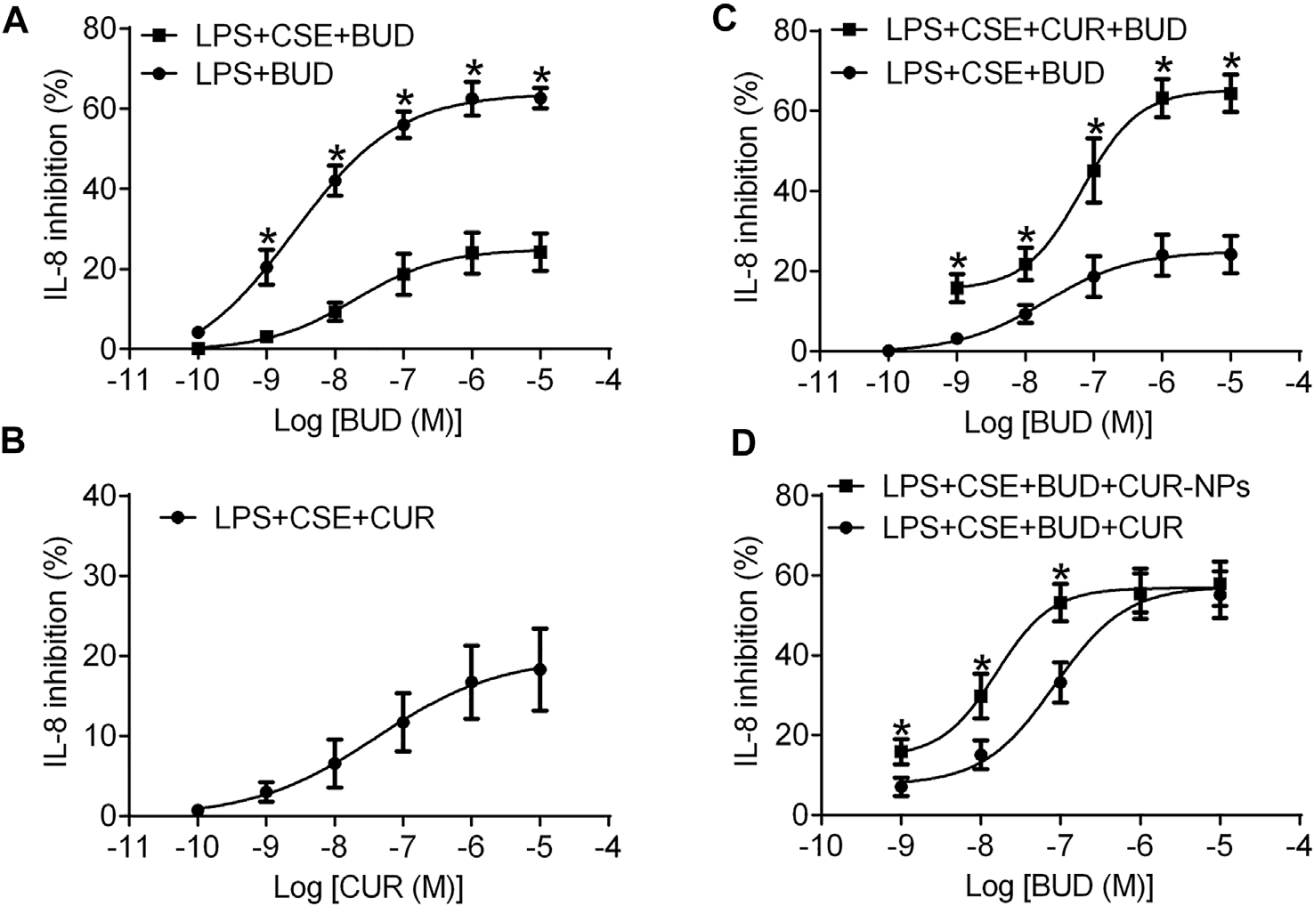
Concentration dependent inhibitory effect of BUD or CUR on IL-8 release induced by LPS alone or in combination with CSE in RTE cells. **(A)** RTE cells were treated with BUD (10^–10^–10^–5^ M) for 15 min and then stimulated with LPS (1 μg/ml) alone or in combination with 10% CSE. **(B)** RTE cells were treated with Cur and then stimulated with LPS (1 μg/ml) in combination with 10% CSE. **(C)** RTE cells were treated with BUD alone or in combination with Cur (100 nM) and then stimulated with LPS in combination with 10% CSE. Cell supernatants were collected after 24 h of cell stimulation and were used for IL-8 assays. *p < 0.05 related to cells incubated with BUD alone in the stimulation of CES and LPS. **(D)** Concentration dependent inhibitory effect of BUD in combination with CUR (100 nM) or Cur-mPEG-PLGA-NPs (equivalent to 100 nM CUR) on IL-8 release induced by LPS in combination with 10% CSE in RTE cells. ^*^ *p* < 0.05 related to cells incubated with BUD and CUR in the stimulation of CES and LPS.

In RTE cells, BUD concentration-dependently inhibited the secretion of IL-8 induced by LPS (Figure 6A; Table 1). In contrast, after being stimulated by LPS and 10% CSE, RTE cells exhibited resistance to the anti-inflammatory effect of BUD (Figure 6A). The maximal inhibition percentage of BUD in RTE cells stimulated by LPS and 10% CSE was significantly lower than that in RTE cells stimulated by LPS alone. The half-inhibitory concentration of BUD showed a corresponding increase in RTE cells stimulated by LPS and 10% CSE (Table 1). The effect of curcumin on BUD resistance induced by 10% CSE was then investigated. Curcumin weakly reduced the LPS- and 10% CSE-induced IL-8 secretion (Figure 6B; Table 1). However, the combination of BUD and 100 nM curcumin achieved a higher maximum inhibition for LPS- and 10% CSE-induced IL-8 secretion than that for BUD alone (Figure 6C; Table 1). The half-inhibitory concentration of BUD in combination with curcumin was lower than that of BUD alone (Table 1).

**TABLE 1:** Maximal efficacy and −log IC_50_ of BUD on IL-8 release.

B	RTE cells	
	Maximal inhibition (%)	−log IC_50_
LPS + BUD	63.94 ± 1.48	8.60 ± 0.14
LPS + CSE + BUD	33.43 ± 1.24^a^	6.77 ± 0.08^a^
LPS + CSE + CUR + BUD	64.30 ± 1.74^b^	7.33 ± 0.10^b^

a p < 0.05 related to cells incubated with BUD alone in the stimulation of LPS.

b p < 0.05 related to cells incubated with BUD alone in the stimulation of CES and LPS.

### Ability of Cur-mPEG-PLGA-NPs to Reverse Budesonide Resistance

The ability of curcumin and Cur-mPEG-PLGA-NPs to reverse budesonide resistance in RTE cells was compared. BUD in combination with Cur-mPEG-PLGA-NPs at doses equivalent to curcumin showed higher inhibitory rates for LPS- and 10% CSE-induced IL-8 secretion than that in combination with 100 nM curcumin (Figure 6D; Table 2). The obtained results demonstrated that curcumin loaded in nanoparticles was more effective in reversing budesonide resistance than curcumin.

**TABLE 2:** Inhibitory effect of BUD in combination with CUR (100 nM) or Cur-mPEG-PLGA-NPs at a dose equivalent to 100 nM CUR on IL-8 release in RTE cells.

	Inhibition (%) of BUD (nM)		
	1	10	100
LPS + CSE + CUR	7.08 ± 2.25	15.08 ± 3.60	33.20 ± 5.06
LPS + CSE + CUR-NPS	15.92 ± 3.18^a^	29.82 ± 5.59^a^	53.26 ± 4.70^a^

a p < 0.05 related to cells incubated with BUD and CUR (100 nM).

### Effect of Curcumin and Cur-mPEG-PLGA-NPs on Budesonide Resistant Markers Altered by Cigarette Smoke

**FIGURE 7:**
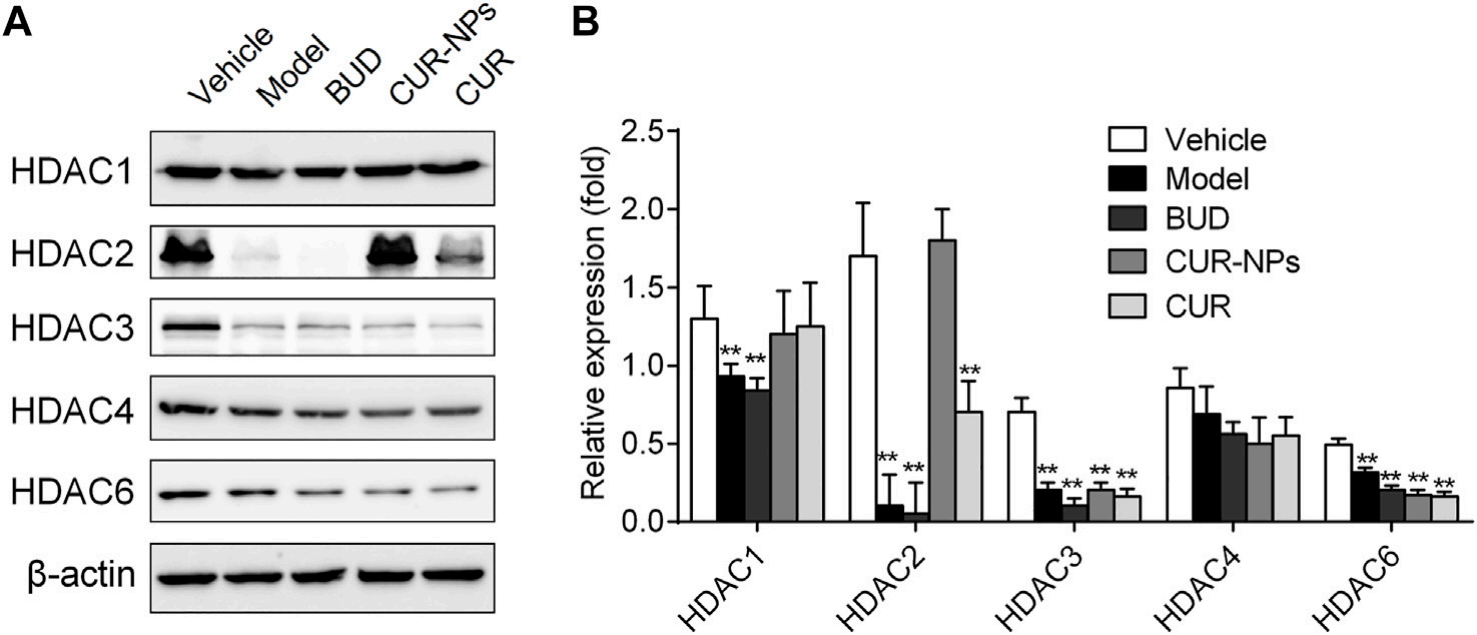
Effects of CUR and Cur-mPEG-PLGA-NPs on HDACs level. **(A)** RTE cells were incubated with BUD (0.1 μM), Cur (1 μM), and Cur-mPEG-PLGA-NPs (equivalent to the concentrations of CUR) for 15 min and stimulated with 10% CSE. **(B)** HDACs levels were detected by western blotting. ^**^ *p* < 0.05 related to CSE.

In RTE cells, the effects of curcumin and Cur-mPEG-PLGA-NPs on HDACs were determined. Western blot analysis was employed to semi-quantitatively determine protein content levels of HDAC2. The results showed that 10% CSE exposure reduced HDAC2 protein levels, and curcumin and Cur-mPEG-PLGA-NPs restored HDAC2 levels in RTE cells (Figure 7 and Supplementary Material). However, other HDAC members were not found to have a regulatory role in the CSE-mediated budesonide resistant in RTE cells. The results showed that curcumin was able to restore the expression level of HDAC2 and curcumin loaded in mPEG-PLGA-NPs was more effective than curcumin.

## Discussion

Inhaled corticosteroids (ICSs) have been widely used in the treatment of COPD and asthma. However, only 20–25% of severe COPD patients show effective responses to ICSs. In a large proportion of COPD patients, ICSs fail to control symptoms and prevent disease progression and are, therefore, challenged by corticosteroid resistance (Xie et al., 2011). It has been confirmed that reduced histone deacetylase (HDAC)-2 is a major contributor to corticosteroid resistance in COPD patients (Tsai et al., 2012). HDAC2 has the ability to promote histone deacetylation in target gene promoter regions and decrease pro-inflammatory gene transcriptional activity, such as TNF-alpha, IL-8, and IL-1 beta (Ghalandarlaki et al., 2014). In the anti-inflammatory process of corticosteroids, HDAC2 is recruited by activated GR to the promoter region of pro-inflammatory genes and switches off the transcription process (Zhang et al., 2013).

A body of evidence has indicated a decrease in HDAC2 activity in the alveolar macrophages of smokers and patients with COPD, and it is related to disease severity and exacerbation. For example, an *in vitro* study indicated that CSE exposure reduced HDAC2 expression and increased pro-inflammatory cytokine expression, such as IL-8 and TNF-alpha, in human macrophages (Adenuga et al., 2009). In peripheral blood mononuclear cells of patients with COPD and smokers, HDAC activities were decreased and negatively correlated to serum IL-8 levels (Meja et al., 2008). Another study confirmed that the messenger RNA (mRNA) and protein expressions of HDAC2 were also significantly lower in COPD patients and that the reduction in HDAC2 activity correlated with the severity of COPD disease (Lao et al., 2006).

The present study revealed that 10% CSE exposure reduced the level of HDAC2 protein in RTE cells. Consequently, the potency (maximum inhibitory percentage) and efficacy (median effective concentration) of BUD inhibiting IL-8 release were reduced in RTE cells incubated with10% CSE and LPS. Curcumin showed a relatively weak inhibitory effect on IL-8 induced by LPS and 10% CSE in RTE cells. However, by increasing HDAC2 levels, curcumin restored the diminished capacity of BUD to inhibit IL-8 release. These findings suggested that curcumin might reverse the 10% CSE-induced corticosteroid resistance by maintaining the level of HDAC2 protein.

A body of evidence indicates that curcumin has shown great therapeutic benefits in various diseases, including inflammatory disorders, cardiovascular disease, cancer, and Alzheimer’s disease. However, rapid metabolism and systemic clearance from the body contribute to low bioavailability of curcumin, limiting its clinical application. After 12 h of the oral administration of 10 g/d and 12 g/d curcumin in a healthy human, only μg/mL quantities of curcumin conjugates (curcumin glucuronides and sulfates) were found in the serum, and among the eight subjects of study, only one had detectable free curcumin in the serum (Anand et al., 2010).

Nanotechnology is a new field of interdisciplinary investigation, and has been widely used in the diagnosis and treatment of diseases (Saka and Chella, 2020). MPEG and PLGA are two biodegradable materials approved by the United Satates Food and Drug Administration for human use. Block copolymers formed by mPEG and PLGA are often used for the preparation of nanoparticles as drug carrier materials. Some studies have indicated that curcumin-loaded mPEG-PLGA nanoparticles are able to overcome the chemical and physical drawbacks of curcumin and improve its biological activity. Besides, after intravenous administrations *in vivo*, curcumin achieved maximum plasma concentration in 5 min, leading to a short elimination half-time of approximately 1.07 h. In contrast, curcumin delivered by curcumin nanoparticles achieved maximum plasma concentration in 2 h, resulting in a significant increase in t1/2 (of about six-fold) compared with that of curcumin. The results concluded that the curcumin-loaded nanoparticles presented a relative threefold higher bioavailability than that of curcumin. In this study, the cellular uptake of Cur-mPEG-PLGA-NPs and curcumin in RTE cells was quantitatively evaluated by fluorescence spectroscopy. The data demonstrated that the cellular uptake of curcumin-loaded mPEG-PLGA-NPs was statistically significantly higher than that of curcumin in RTE cells. This suggested that polymeric nanoparticles enhance the internalization and retention effects of curcumin. Then, the ability of Cur-mPEG-PLGA-NPs to restore corticosteroid-mediated inhibition on IL-8 was further studied. The data demonstrated that at low doses, BUD in combination with Cur-mPEG-PLGA-NPs represented higher inhibitory rates on IL-8 than that in combination with curcumin in RTE cells incubated with 10% CSE and LPS. These results were consistent with cellular uptake results and indicated that Cur-mPEG-PLGA-NPs offered higher bioavailability in reversing CSE-induced corticosteroid resistance. Further study intends to isolate and culture alveolar macrophages and airway epithelial cells from smoking COPD patients, smokers with normal lung function and normal controls, and use LPS stimulation to investigate the role of curcumin in reversing hormone resistance.

In summary, 10% CSE exposure reduced the potency and efficacy for BUD inhibiting IL-8 release in RTE cells, and induced corticosteroid resistance. Curcumin restored the diminished capacity of BUD to inhibit IL-8 release induced by LPS and 10% CSE. Curcumin loaded in mPEG-PLGA nanoparticles was uptaken more in RTE cells, and exhibited more effectiveness in reversing corticosteroid resistance than curcumin. All these findings indicate that Cur-mPEG-PLGA-NPs have great potential as a therapeutic agent. Both curcumin and Cur-mPEG-PLGA-NPs at high doses restored the decrease in HDAC2 levels caused by 10% CSE exposure. However, at relatively low doses, only Cur-mPEG-PLGA-NPs showed a restoration effect on HDAC2 levels. This explains the greater efficacy of Cur-mPEG-PLGA-NPs in reversing corticosteroid resistance.

## Data Availability

The raw data supporting the conclusion of this article will be made available by the authors, without undue reservation.
