# Supplementary figures and images for: Curcumin-Loaded mPEG-PLGA Nanoparticles Attenuates the Apoptosis and Corticosteroid Resistance Induced by Cigarette Smoke Extract
Source: Front Pharmacol. 2022 Feb 25;13:824652. doi: 10.3389/fphar.2022.824652 (PMC8914114; doi:10.3389/fphar.2022.824652)

HDAC4

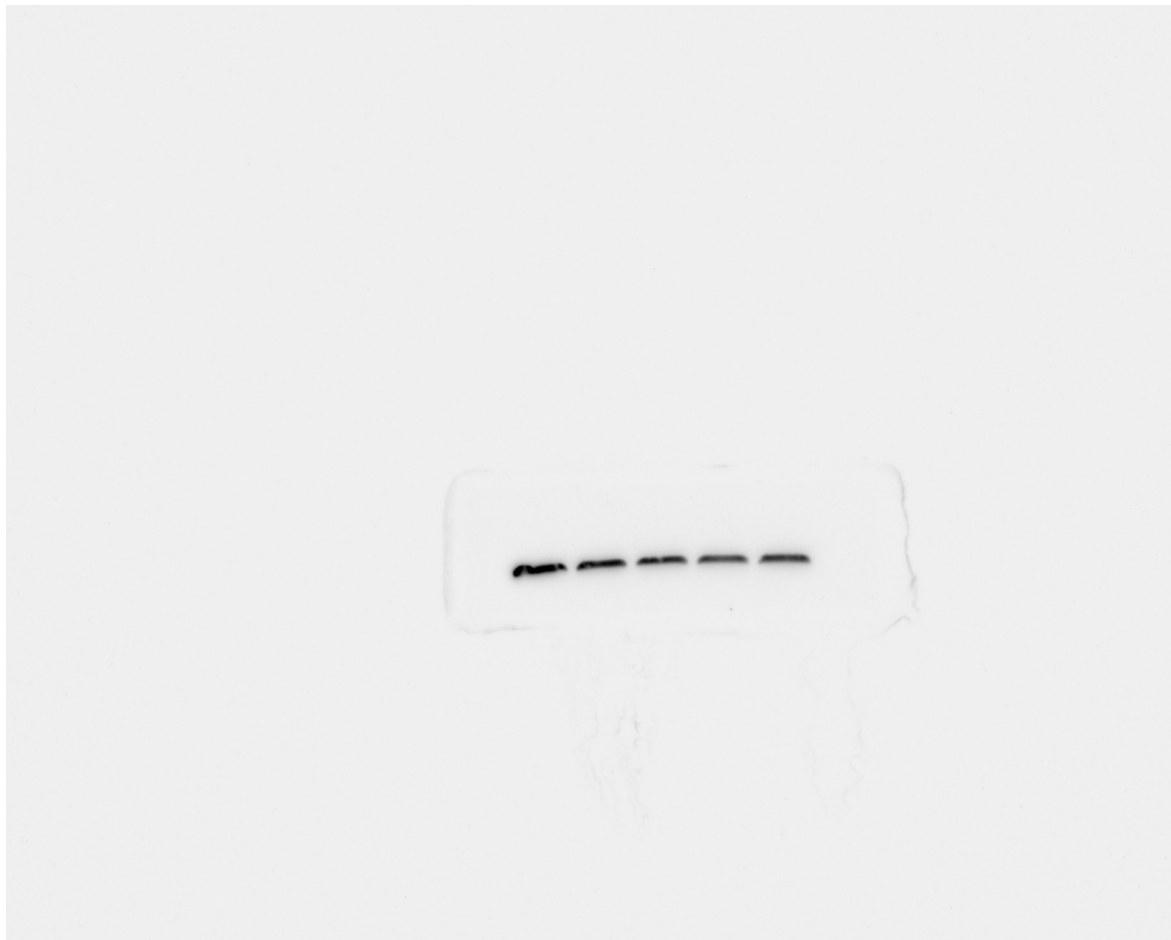

## HDAC3

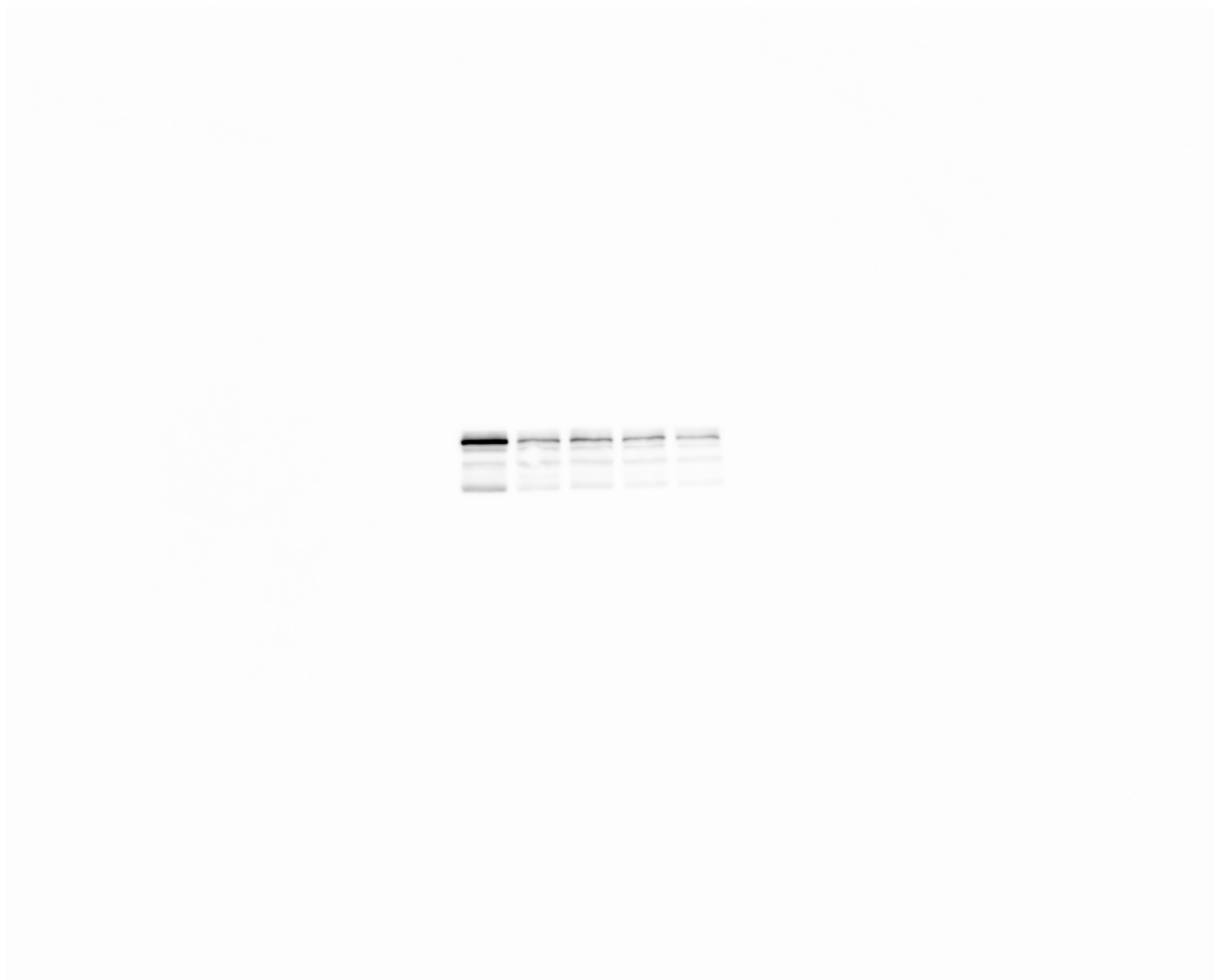

HDAC2

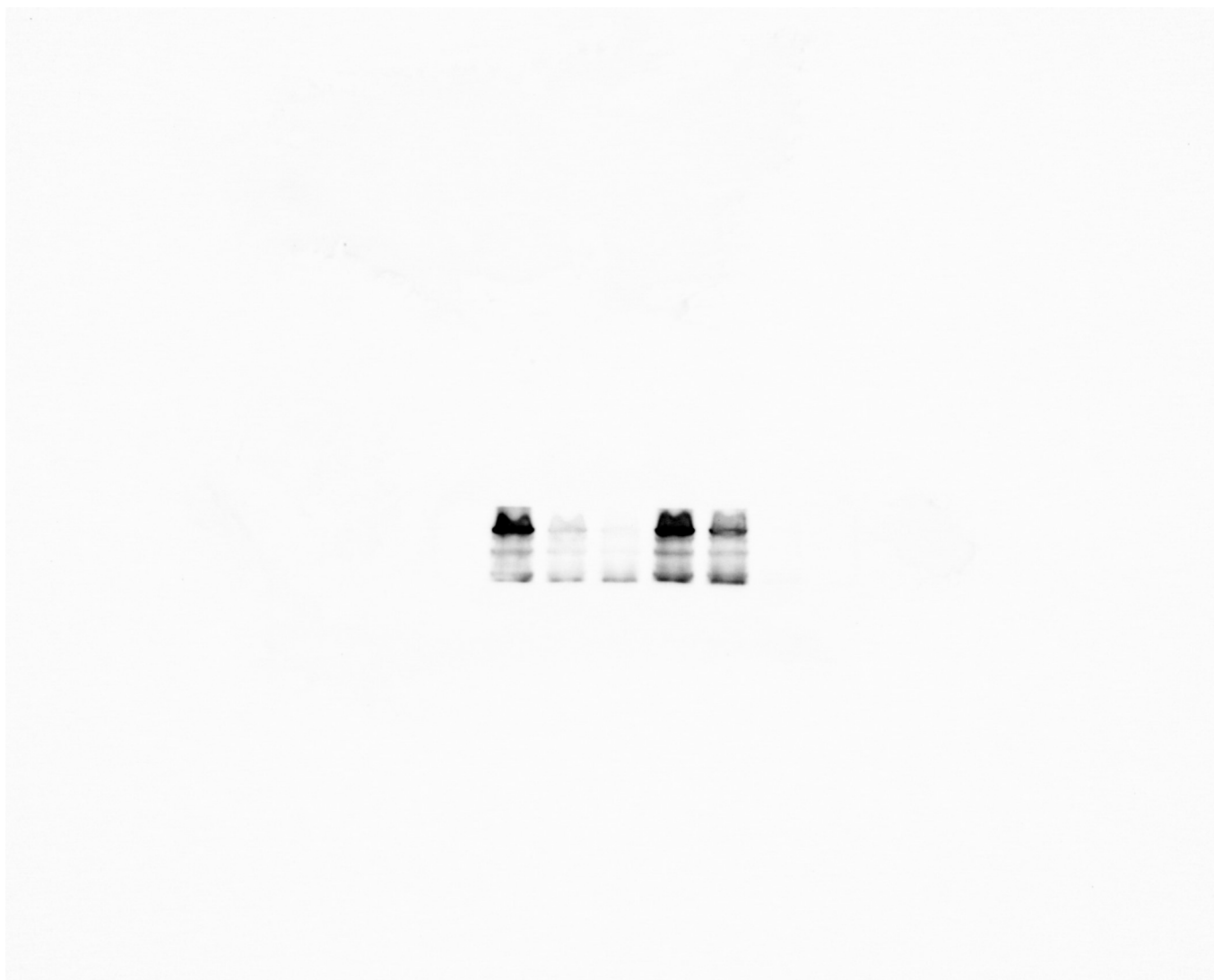

HDAC1

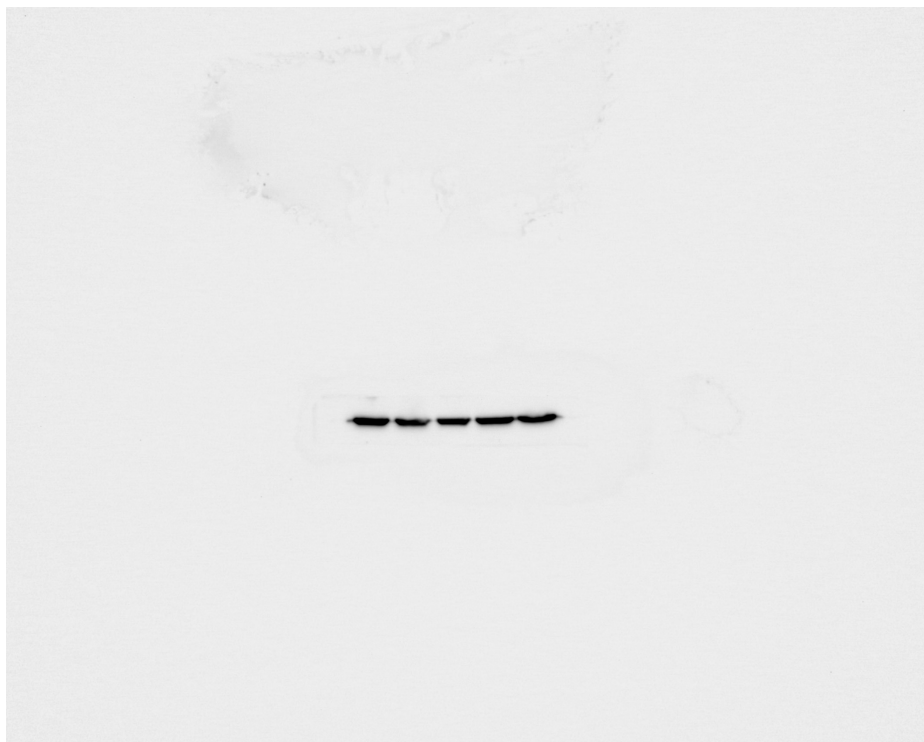

$\beta$ -actin

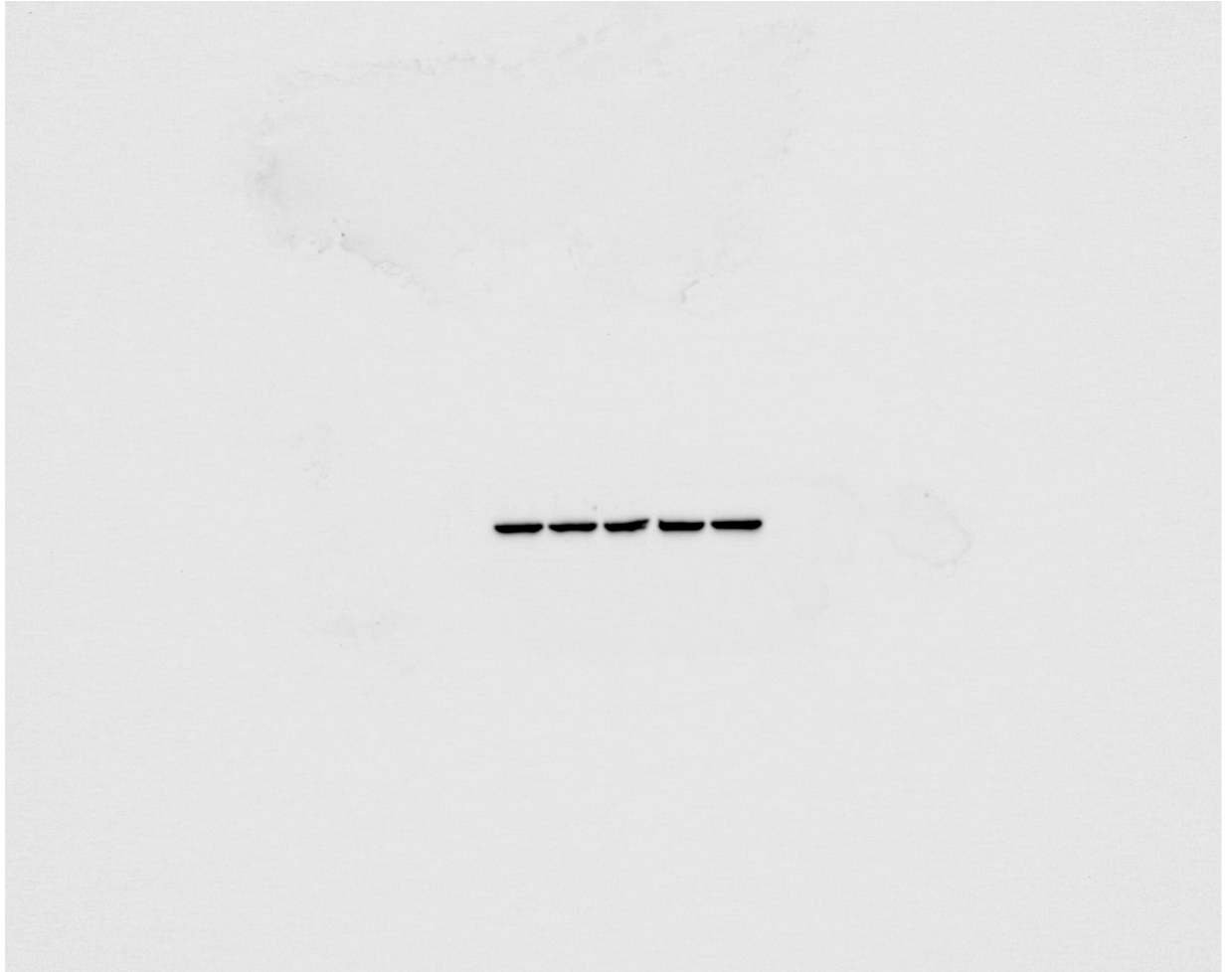

HDAC6

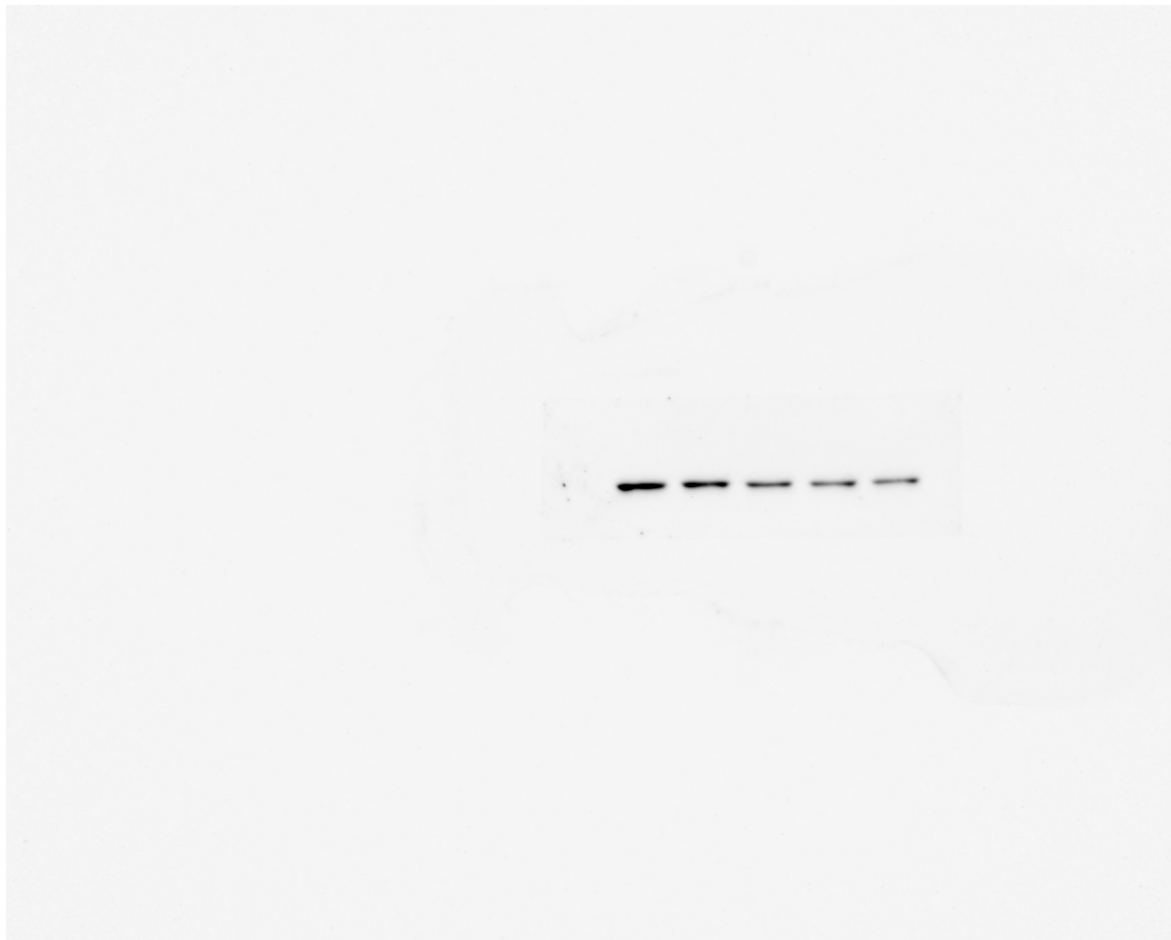

Supplement: Supplementary file 1 [file DataSheet1.PDF]
